# Fusing Wearable Biosensors with Artificial Intelligence for Mental Health Monitoring: A Systematic Review

**DOI:** 10.3390/bios15040202

**Published:** 2025-03-21

**Authors:** Ali Kargarandehkordi, Shizhe Li, Kaiying Lin, Kristina T. Phillips, Roberto M. Benzo, Peter Washington

**Affiliations:** 1 Information and Computer Sciences, University of Hawaii at Manoa, Honolulu, HI 96822, USA; kargaran@hawaii.edu (A.K.); kylin@hawaii.edu (K.L.); 2 Department of Statistics, Stanford University, Stanford, CA 94305, USA; shizheli@stanford.edu; 3 Institute of Linguistics, Academia Sinica, Taipei 11529, Taiwan; 4 Center for Integrated Health Care Research, Kaiser Permanente Hawaii, Honolulu, HI 96817, USA; kristina.t.phillips@kp.org; 5 Department of Health Systems Science, Kaiser Permanente Bernard J. Tyson School of Medicine, Pasadena, CA 91101, USA; 6 Division of Cancer Prevention and Control, Department of Internal Medicine, College of Medicine, The Ohio State University Comprehensive Cancer Center, The Ohio State University Wexner Medical Center, Columbus, OH 43210, USA; roberto.benzo@osumc.edu; 7 Division of Clinical Informatics and Digital Transformation, Department of Medicine, University of California, San Francisco, CA 94143, USA

**Keywords:** mental health, depression, stress, anxiety, wearable, machine learning, study

## Abstract

The development of digital instruments for mental health monitoring using biosensor data from wearable devices can enable remote, longitudinal, and objective quantitative benchmarks. To survey developments and trends in this field, we conducted a systematic review of artificial intelligence (AI) models using data from wearable biosensors to predict mental health conditions and symptoms. Following PRISMA guidelines, we identified 48 studies using a variety of wearable and smartphone biosensors including heart rate, heart rate variability (HRV), electrodermal activity/galvanic skin response (EDA/GSR), and digital proxies for biosignals such as accelerometry, location, audio, and usage metadata. We observed several technical and methodological challenges across studies in this field, including lack of ecological validity, data heterogeneity, small sample sizes, and battery drainage issues. We outline several corresponding opportunities for advancement in the field of AI-driven biosensing for mental health.

## 1. Introduction

### 1.1. Background

By 2030, global mental health expenditures are anticipated to exceed USD 16 trillion [1]. Despite this, mental health care has generally received less attention and priority compared to the treatment of physical and physiological health conditions [2,3]. Mental health disorders have a global prevalence rate exceeding 10%, affecting approximately 450 million individuals [4,5]. Depression and anxiety disorders are particularly common, and although the number of U.S. adults who seek treatment for these conditions has increased, many adults with mental health diagnoses do not seek services. This is often due to a number of barriers, such as the cost of treatment [6]. Thus, alternative approaches to care are needed [7].

One way to improve the management of mental health symptoms is to continuously monitor them using physiological health signals. Wearable devices such as smartwatches and fitness bands often come equipped with an array of integrated sensors, spanning communication technologies (like WiFi and Bluetooth), motion sensors (such as accelerometers and gyroscopes), biosensors (including heart rate monitors and electrodermal activity sensors), and environmental sensors (such as ambient pressure and temperature sensors), among others. Though the application of such sensors has potential, there are several challenges to their real-world use in research and translation, especially concerning the reliability of the data [8,9,10].

Biosensors were first developed for medical use in controlled clinical environments such as intensive care units. In 1962, Leland C. Clark and Champ Lyons introduced the first biosensor, an amperometric enzyme electrode for glucose detection [11]. Advancements in miniaturization, wireless communication, and computational processing have since enabled the transition of biosensors into wearable formats suitable for everyday use [12].

Over the past decade, the integration of AI with biosensing technologies has led to a dramatic increase in the utility of biosensors for healthcare. AI-powered biosensors can process and analyze large volumes of physiological data in real time, enabling the identification of subtle patterns associated with a variety of conditions, including but not limited to adverse mental health events. Wearable biosensors provide multimodal physiological and behavioral data while AI models process these data into meaningful insights [13,14]. For instance, machine learning (ML) algorithms have been applied to detect stress from heart rate variability, identify depression through changes in physical activity, and infer emotional states using electrodermal activity [15]. We conduct a systematic review to assess current practices and to identify ongoing challenges and corresponding research opportunities in the field of AI-driven biosensing for mental health.

### 1.2. Objectives

The primary objective of this systematic review is to evaluate the current state of research on the integration of wearable-based biosensors with ML for mental health monitoring. We aim to identify key trends, challenges, and gaps in the field. Because it is infeasible to review every possible mental health condition, we focus our review primarily on research targeting depression, stress, and anxiety monitoring. Given the high prevalence of these conditions, we expect that findings for these conditions will generalize to a plethora of other conditions within psychiatry and the behavioral sciences more broadly.

We sought to answer the following specific research questions in our review:

- What types of biosensors and data collection methods are most commonly used for AI-based monitoring of mental health conditions such as depression, stress, and anxiety?
- What methodological challenges and gaps exist for research at the intersection of biosensing, AI, and mental health?

## 2. Materials and Methods

We systematically searched and analyzed the relevant literature from computing, technology, and medical databases (Table 1), adhering to PRISMA guidelines. Our search strategy incorporated a range of terms related to mental health and wellbeing, wearable biosensing, and AI strategies to ensure we reviewed studies involving all three of these aspects. We also targeted specific biosignals with keywords such as “heart rate”, “HRV”, “GSR”, and “ECG” to focus on physiological data collection. We included terms like “stress”, “anxiety”, “depression”, and “wellbeing” to constrain our search. While this is clearly not a comprehensive list of mental health conditions or biosignals, we decided to focus on these common conditions and biosignals as demonstrative examples that can help us glean methodological insights. In Table 1, we detail the specific keyword queries we used in Web of Science to identify relevant studies focused on wearable technology, biosignals, and mental health.

We included studies that met the following criteria: (1) they were empirical in nature; (2) they did not rely on publicly available datasets but instead generated new datasets through participant recruitment; (3) they primarily examined participants’ mental health and/or wellbeing; (4) they collected biosensor data using wearable devices; and (5) they described at least some of the data collection procedures as passive or requiring minimal user interaction. We considered English-language studies published up to August 2024. We included only peer-reviewed journal articles and conference proceedings, excluding extended abstracts and review papers.

We excluded studies that relied on publicly available datasets for analysis as well as studies with a sole focus on mobile devices rather than wearables. We also excluded studies that did not incorporate any AI strategies for predicting, estimating, or monitoring mental health issues and were only limited to data analysis.

The first two authors filtered papers and applied the inclusion and exclusion criteria. We discussed disagreements until we reached a consensus. We included a number of unique papers from each database (Table 1), with IEEE yielding the highest number (13 papers), followed by MDPI (11), Elsevier (6), JMIR (6), and Frontiers (3).

## 3. Results

**Figure 1:**
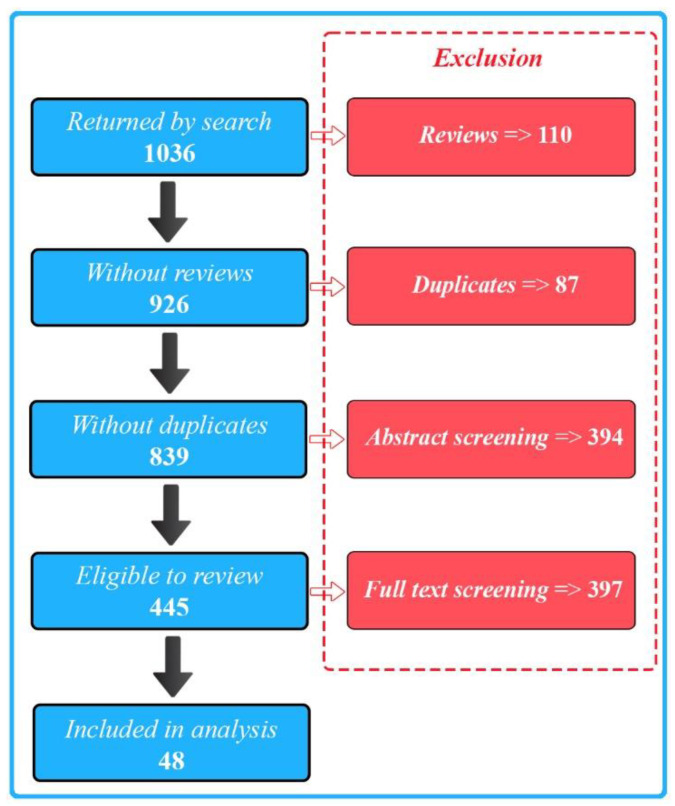
PRISMA flow diagram illustrating the study selection process.

We selected a total of 48 publications for our final full review. We identified these from 1036 initial search results (Figure 1) and excluded the majority of the papers due to a lack of either biosensing, wearable, or AI aspects of the described research.

**Figure 2:**
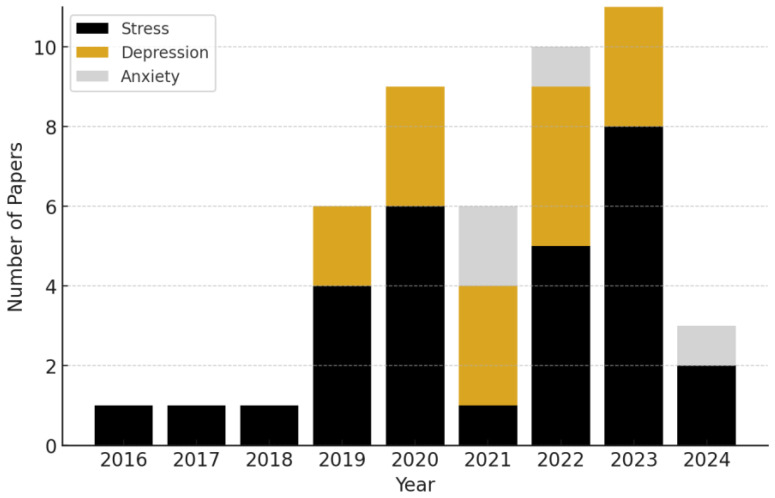
Annual number of publications, categorized by mental health condition, from 2016 to 2024. We observe a continually upward trend in the number of mental health biosensing publications incorporating AI.

We present the categorization of research papers by the specific mental health conditions they address, per year, in Figure 2. The data reveal a clear upward trend in the volume of research integrating wearables and AI over time, particularly starting in 2019.

We found that stress was the most frequently studied condition out of our reviewed sample, representing ~60.0% of the studies. Depression followed as the second most common focus, accounting for ~31.0% of the research. Anxiety comprised ~9% of the reviewed studies.

We list and describe the studies broken down by each of our target conditions in Table 2, Table 3 and Table 4. For each study, we detail the datasets, sample size, and features extracted from biosensing devices. We also discuss the AI strategies used and the resulting performance metrics. We summarize the key contributions, novelties, and research gaps identified in each paper.

### 3.1. Stress

**Figure 3:**
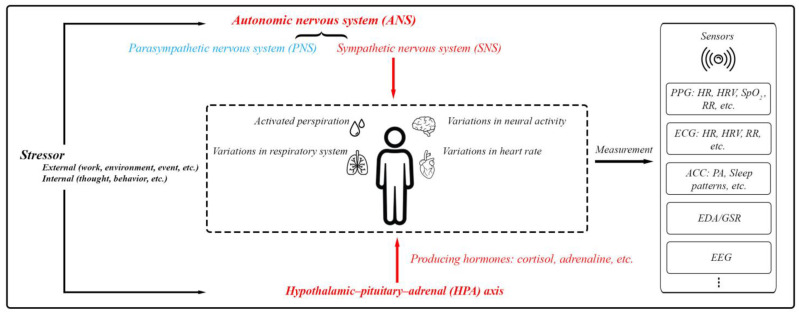
The body’s physiological responses to stress and the standard methods used for assessing these stress responses. This includes PPG (photoplethysmography) for measuring blood volume changes, heart function, and respiratory rate (RR); ECG (electrocardiography) for measuring heart function and RR; ACC (accelerometer) mainly for physical activity (PA) and sleep patterns; EDA/GSR for skin conductivity; and EEG (electroencephalography) for brain activity.

Stress involves the activation of the autonomic nervous system (ANS) and the hypothalamic–pituitary–adrenal (HPA) axis. The ANS, comprising the sympathetic nervous system (SNS) and parasympathetic nervous system (PNS), manages body functions to maintain balance. During stress, the SNS heightens responses like heart and respiration rates as well as sweat production. Simultaneously, the HPA axis triggers cortisol secretion, which supports the body’s fight-or-flight response by modifying physiological processes and reinforcing SNS effects [8,9,10,16]. Monitoring the activities of the SNS and HPA axis can offer insights into a person’s overall health status, including their mental well-being (Figure 3).

We present an overview of the studies focused on stress in Table 2. We outline the specific details of each empirical study, including sample size, wearable device and biosignals used for analysis, AI methodologies, model performances, data collection environment, monitoring details, and identified research challenges.

**Table 2:** Biosensing papers reviewed focusing on stress. The data collection environment (Env.) is specified as IL (In-Lab) or IW (In-the-Wild).

Article/Year Application	Sample Size	Wearable/ Feature Space	Prediction Task/ AI Details	Env.	Labels/ Monitoring Details	Research Challenges
(Akubulut et al., 2020) [17] Real-time stress monitoring and management	30	❖ CVDiMo wearable sensor device 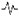 ECG, GSR/EDA, SpO2, blood pressure, glucose, body temperature readings	❖ Multiclass classification ● A feed-forward neural network (FFNN) with 2 hidden layers and linear output ● 92% (metabolic syndrome) and 89% (others) accuracy	IL	● Labels: self-reported emotional ratings (1–10) after emotion-inducing videos ● 6 labels/participant (one for each video) ● Monitoring: approximately 1 h	● GSR/EDA biases challenge accuracy ● Small sample size limits generalizability ● Larger datasets needed for robust, broader analysis
(Anusha et al., 2019) [18] Pre-surgery stress detection	41	❖ ADI-VSM device 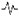 EDA/GSR	❖ Multiclass classification ● Localized supervised learning with adaptive dataset partitioning ● 97.83% accuracy; 85.06% without person-specific factors	IL	● Labels: State–Trait Anxiety Inventory (STAI) and salivary cortisol levels ● 2 STAI surveys assessed stress levels (low, moderate, and high) ● Monitoring: approximately 3 h before surgery	● Stress response variability limits applicability ● Clinical conditions affect detection accuracy ● Small, surgery-specific sample size
(Aristizabal et al., 2021) [19] Wearable and self-report stress detection	18	❖ Empatica E4 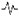 Heart rate (HR) derived from PPG, EDA/GSR, and skin temperature (ST)	❖ Binary classification ● Supervised deep neural networks with logistic generalized estimating equation (GEE) for stress estimation ● 96% accuracy (wearable + survey); 88% accuracy (wearable only)	IL	● Labels: self-reported stress/anxiety (STAI and PSS-10) and salivary cortisol ● 8 labels/participant: 4 cortisol samples; 4 self-reports ● Monitoring: 2 h monitoring with 4 prompts (baseline, post-stress, and recovery)	● Small sample size ● Imbalanced data labels ● Data showed high variability ● Research-grade wearable limits translatability ● Stress detection focused on induced stress in controlled settings
(Barki and Chung, 2023) [20] Mental stress detection	14	❖ In-ear device (earbod) PPG system 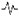 PPG, accelerometer (ACC)	❖ Binary classification ● Supervised convolutional neural network (CNN) ● 92.04% accuracy, 90.80% F1-score; 96.02% accuracy with white-Gaussian noise	IL	● Labels: stress/no-stress classifications based on PPG signals and mental stress tasks (Stroop test; arithmetic) ● 2 labels/participant (stressed and non-stressed) ● Monitoring: 3 min per task	● Small sample size limits generalizability ● Limited stressors reduce real-world applicability ● Further research needed for diverse settings and populations
(Betti et al., 2017) [21] Stress detection via physiological signals correlated with salivary cortisol using PCA analysis for early warnings	15	❖ Zephyr BioHarness 3 (chest belt for HRV), Shimmer Sensor (EDA/GSR monitoring), and MindWave Mobile EEG headset (EEG) 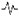 HRV, EDA/GSR, EEG	❖ Binary classification ● Supervised ML ● 84% sensitivity, 90% specificity, and 86% overall accuracy	IL	● Labels: salivary cortisol levels during the Maastricht Acute Stress Test (MAST) ● 5 salivary cortisol samples and multiple physiological data points per participant ● Monitoring: approximately 1 h	● Small sample size limits generalizability ● Offline processing hinders real-time application ● MAST protocol complexity and cultural adjustments affect reproducibility
(Booth et al., 2022) [22] Predicting perceived stress	606	❖ Garmin Vivosmart 3 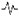 HR derived from PPG and ACC for physical activity (PA)	❖ Binary classification ● Supervised ML; multimodal ● 62% accuracy, 65% precision, and 89% recall	IW	● Labels: daily self-reported perceived stress using a 5-point Likert scale ● 56 labels/participant (daily for 56 days) ● Monitoring: approximately 2 months ● Prompting: 1–3 times daily via SMS (at 8 am, noon, or 4 pm) to complete the surveys	● Low resolution limits daily stress assessment ● More contextual data on activities and environment needed ● Inadequate data limits GRU/LSTM; stronger temporal models required ● Non-compliance and contextual variability impact predictions
(Campanella et al., 2023) [23] Stress detection	29	❖ Empatica E4 bracelet 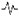 HRV derived from PPG and EDA/GSR	❖ Binary classification ● Supervised ML ● 71% precision, 60% recall, and 65% F1-score (random forest (RF))	IL	● Labels: stress/no-stress classifications during cognitive, social, and physiological stress tasks ● 2 labels/participant (stressed and non-stressed) ● Monitoring: approximately 15–20 min per task	● Unbalanced data impacts classifier performance ● Small sample size and lab setting limit generalizability ● Noise from Empatica E4 and lack of real-life stressors reduce accuracy ● Larger, diverse samples and varied stressors needed for robustness
(Can et al., 2019) [24] Real-life stress monitoring system during a programming contest	21	❖ Samsung Gear S1, S2, and S3 (HRV) and Empatica E4 (EDA/GSR, ST, ACC) 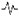 HRV derived from PPG, EDA/GSR, ACC, and ST	❖ Multiclass classification ● Supervised ML ● 86–92% accuracy (separate vs. three-model); 88–97% accuracy (general vs. person-specific models)	IL	● Labels: self-reported stress levels using the STAI ● 2 stress-related labels/participant (before and after the stress task) ● Monitoring: approximately 30 min	● Device-related data quality variations impact accuracy ● Inconsistencies from subjective self-reported stress ● Future efforts focus on better devices and refined models for subjective stress data
(Can et al., 2020) [25] Smartwatch-based stress detection validated with primary school teachers	32	❖ Samsung Gear S1, S2 (HRV), and Empatica E4 (EDA/GSR, ST, and ACC) 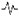 HRV derived from PPG, EDA/GSR, ACC, ST, and IBI	❖ Multiclass classification ● Personalized models ● Up to 94.44% accuracy (HR signal); 100% accuracy (EDA/GSR signal)	IL	● Labels: perceived stress (NASA-TLX) during lecture, exam, and recovery ● 3 labels/participant ● Monitoring: approximately 1.5 to 2 h ● Prompting: after each session to complete surveys	● Lower smartwatch data quality mitigated by artifact detection ● Notifications risk increasing stress without interventions ● Limited generalizability due to specific participant group and setting
(Chen and Lee, 2023) [26] Stress measured in students using Sudoku in distracting environments for real-time educational assessment	30	❖ Polar Verity Sense (PPG), BMD101 (ECG), and NeuroSky MindWave Mobile 2 (EEG) 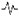 PPG, ECG, and EEG	❖ Multiclass classification ● StressNeXt Model, Attention-LRCN, and Self-Supervised CNN ● 95–99% accuracy; 93–96% F1-score	IL	● Labels: self-reported stress (3-point scale) after Sudoku in noisy, monitored, and comforting scenarios ● 3 labels/participant (one for each scenario) ● Monitoring: approximately 45 min (15 min per Sudoku puzzle scenario)	● Self-reported stress bias affects reliability ● Adding GSR/EDA and respiratory signals can improve accuracy ● Larger datasets needed for robustness and broader applicability
(Golgouneh et al., 2019) [27] Portable stress monitoring system for continuous stress index (SI) estimation and classification	37	❖ Custom-fabricated wearable devices featuring PPG and GSR/EDA sensors developed by the RCDAT 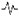 PPG and GSR/EDA	❖ Multiclass classification ● Classical machine learning: K-nearest neighbor (KNN), ANNs, Naive Bayes (NB), and support vector machine (SVM) used for stress classification ● Best performance: KNN (K = 3) with 85.3% accuracy	IL	● Labels: self-reported stress/relaxation levels on a scale of 0 to 4, completed by participants after each test ● 3 labels/participant (relaxed, normal, and stressed) ● Monitoring: approximately 30 min during the stress-inducing tasks	● Limited generalizability due to sensor types, conditions, and participant age range ● Validation needed on diverse datasets and sensors ● Computational complexity for real-time use not fully evaluated
(Halim and Rehan, 2020) [28] Wearable system identifies driver emotions using hemispheric asymmetry to link brain dynamics with stress	86	❖ EMOTIV EPOC+ EEG headset, a 16-channel device 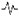 EEG spectral power analyzed across theta, alpha, beta, and gamma bands	❖ Binary classification ● SVM, ANN, and RF evaluated using precision, sensitivity, specificity, F-measure, and G mean ● 97.95% accuracy, 89.23% precision, and 94.92% specificity	IL	● Labels: self-reported emotional states (stress/relaxation) based on the Self-Assessment Manikin (SAM) after driving sessions ● 2 labels/participant (relaxed and stressed) ● Monitoring: approximately 30 min per session	● Excludes fatigue, drowsiness, and alcohol effects ● Self-reports as ground truth introduce subjectivity ● Lab setting limits real-world applicability
(Bin Heyat et al., 2022) [29] Monitoring researchers’ mental stress with an automatic stress detection system	20	❖ A smart T-shirt developed by Hexin Medical Co. Ltd., featuring silver-coated flexible dry electrodes 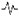 ECG (single lead)	❖ Binary classification ● Decision Tree (DT), NB, RF, and Logistic Regression (LR) to classify the intra-subject (mental stress and normal) and inter-subject classification ● Intra-subject: 93.30% accuracy, 96.70% specificity, and 93.50% F1-score; inter-subject: 94.10% accuracy	IL	● Labels: self-reported emotional states (stress/relaxation) using the SAM and anxiety levels using STAI ● 2 labels/participant (one for each driving session: relaxed and stressed) ● Monitoring: approximately 30 min per session	● Data from 20 subjects limits generalizability ● Single-signal focus overlooks multi-signal interactions ● Small, homogeneous sample risks overfitting ● Unspecified gender distribution affects generalizability
(Kikhia et al. 2016) [30] Detecting stress in dementia patients, with personalized stress threshold settings	6	❖ Philips DTI-2 wristband sensor 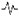 GSR/EDA and ACC	❖ Binary classification ● Base learners: neural networks, splines, ridge regression, RF, GLMs, Gaussian process, XGBoost, KNN, and SVM ● Four-fold cross-validation used to predict anxiety symptom changes between MIDUS-1 and MIDUS-3 ● 89% accuracy at the highest threshold	IW	● Labels: stress monitored via wearables; caregiver observations as ground truth ● 2 labels/participant (stressed/not stressed) based on the analysis of physiological data and clinical observations ● Monitoring: 2 months	● Limited participants reduce generalizability ● GSR/EDA-only algorithm with five severity levels oversimplifies stress ● Additional physiological data needed for better accuracy
(Kim et al., 2020) [31] Mental stress assessment and monitoring	21	❖ EGG: BIOPAC EGG100C with disposable electrodes ECG and RESP: BIOPAC BN-RSPEC and BN-RESP-XDCR with elastic bands 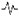 Electrogastrogram (EGG), ECG, and respiratory signals (RESP)	❖ Multiclass classification ● Conventional machine learning models: SVM, LR, RF, DT, and KNN were used as classifier models ● 70.15% accuracy; 0.741 AUC	IL	● Labels: perceived stress (10-point VAS) before and after tasks ● 2 labels/participant ● Monitoring: approximately 50 min during the relaxation and stress-inducing tasks (arithmetic and Stroop tasks)	● Small sample size limits the generalizability ● Protocol-based categorical stress levels restrict analysis depth ● Wired equipment limits real-time monitoring
(Nath et al., 2022) [32] Stress classification framework for older adults	19	❖ Shimmer3 GSR+ 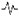 EDA/GSR and BVP	❖ Binary classification ● DT, KNN, and Probability- and Kernel-based classifier ● 0.95 F1-Micro, 0.87 Macro, and 0.81 AUC	IL	● Labels: salivary cortisol levels recorded during the Trier Social Stress Test (TSST) ● 5 labels/participant (based on cortisol samples at different stages: baseline, after stress induction, and recovery) ● Monitoring: approximately 1 h	● Cortisol-only stress definition may miss rapid indicators ● Continuous EEG is impractical for older adults, risking frustration and anxiety
(Perez et al., 2018) [33] Stress estimation on students in controlled environments like classrooms	12	❖ COTS wrist wearable devices equipped with HR, GSR/EDA, ST, and ACC sensors 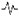 HR, ST, GSR/EDA, and ACC	❖ Multiclass classification ● Supervised ML models ● 99–100% accuracy	IL and IW	● Labels: self-reported stress (5-point Likert) after each task ● 6 labels/participant across lab and classroom phases ● Monitoring: approximately 30–40 min per session over multiple sessions	● Subject variability and device differences hinder replication ● Wearables outside the classroom could capture greater physiological variation over time
(Rescio et al., 2023) [34] Non-invasive stress detection in industrial settings	20	❖ Shimmer GSR 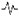 PPG, EDA/GSR, and Ambient sensor	❖ Binary classification ● Supervised (DT, RF, and KNN) and unsupervised (K-means, GMM, and SOM) algorithms evaluated ● GMM achieved 77.4% accuracy (one level) and 75.1% (two levels)	IL	● Labels: self-reported stress (5-point scale) after each task ● 4 labels/participant (one after each stress-inducing task) ● Monitoring: approximately 40 min during stress tasks (Trier Social Stress Test, Stroop Test, and Math Test)	● Lab conditions may not reflect real industrial complexities ● Unsupervised learning had lower accuracy than supervised methods
(Ribeiro et al., 2023) [35] Five-level stress classification	16	❖ MAX30102 PPG sensor for HR, HRV, and SpO2, Grove GSR sensor for GSR/EDA, and an infrared sensor for skin temperature 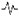 GSR/EDA and PPG (HR, HRV, RR, and SpO2)	❖ Multiclass classification ● Fuzzy logic ● 75% sensitivity, 97% specificity, and 93% accuracy	IL	● Labels: self-reported stress (5-point scale) after thermal stress phases ● 5 labels/participant (rest, cold stress, recovery, heat stress, and final recovery) ● Monitoring: approximately 27 min	● Thermal stress focus limits generalization ● System complexity and size hinder daily use ● Costly hardware reduces accessibility ● Binary relevance overlooks stress indicator correlations
(Sevil et al., 2020) [36] Real-time detection of physical activity and acute psychological stress	24	❖ Empatica E4 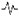 ACC, HR, BVP, GSR/EDA, and ST	❖ Multiclass classification ● Ensemble models ● 99.30% PA accuracy; 92.70% APS accuracy. 89.90% accuracy for simultaneous occurrences of both PA and APS	IL	● Labels: self-reported stress levels ● 207 experiments with stress labels from physical and psychological tasks (e.g., treadmill, bike, and mental/emotional stress) ● Monitoring: 20–60 min per experiment	● Limited data volume favored simpler ML techniques over advanced deep learning models ● Larger datasets are needed in future research to effectively train and evaluate deep learning models
(Tonacci et al., 2020) [37] Predicting stress reduction following relaxation at workplace	24	❖ Shimmer 3 GSR+ 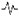 ECG (HRV) and GSR/EDA	❖ Binary classification ● Matlab “Classification Learner” trained classifiers on autonomic features from ECG and GSR/EDA ● 79.2% accuracy	IL	● Labels: self-reported anxiety (STAI; VAS-A) before and after relaxation ● 2 labels/participant ● Monitoring: 12 min (baseline, audio–video relaxation, rest, and video/audio-only relaxation)	● Predominantly female sample limits generalizability ● Small sample size restricts demographic analysis and model training ● Short protocol duration limits autonomic changes ● Non-optimized YouTube media may affect intervention efficacy
(Toshnazarov et al., 2024) [38] Accurate stress detection in natural settings	IL: 26 IW: 28	❖ Samsung galaxy Watch 5, Polar H10 chest strap 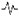 HRV from PPG, HR, IBI, and Tri-axial ACC	❖ Binary classification ● The ML models include AdaBoost, GB, LR, MLP, RF, SVM, and extreme GB (XGBoost) ● 84% F1-score (lab); 71% F1-score (real-life)	IL and IW	● Labels: self-reported stress ecological momentary assessments (EMA) via smartphones ● 12 labels/participant daily, randomized intervals ● 2-week continuous monitoring (Samsung smartwatch, and smartphone) ● Lab stress tasks: public speaking (9 min), cognitive (8 min), and physical (4 min), with 30 min rest ● Prompting: 12 times/day for stress and contextual data	● Daily-life variability may affect SOSW performance ● Small sample size limits generalizability; larger data needed ● Continuous sensing may impact smartwatch battery life ● Data transmission issues could disrupt capture ● Results depend on smartwatch model; sensor accuracy may vary
(Tutunji et al., 2023) [39] Passive stress detection, supporting personalized psychiatry	83	❖ Empatica E4 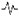 HR, skin conductance (SC), ST, and ACC	❖ Binary classification ● Generalized linear mixed-effects models (GLMM) with maximal fitting ● Individualized (leave-one-beep-out) and group-based (leave-one-subject-out) models tested ● 29.87% error rate	IW	● Labels: self-reported stress and affect (EMA, 7-point Likert) ● 6 labels/day per participant (exam and control weeks) ● Monitoring: 2-week continuous ● Prompting: EMA surveys prompted 6 times/day	● Cross-sectional design limits long-term predictions ● Low accuracy due to real-life stress detection challenges ● Device reliability affected by data noise in daily use ● Small sample and student exam focus limit generalizability
(Umer, 2022) [40] Monitoring physical and mental stress in construction workers	8	❖ Equivital EQ02 Life Monitor vest 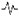 HR and HRV derived from ECG, ST, breathing rate (BR), and SC	❖ Multiclass/binary classification ● RUS Boosted Trees, Subspace KNN, and gagged trees ● 94.7% accuracy for simultaneous physical and mental stress monitoring	IL	● Labels: self-reported physical (RPE) and mental stress (NASA-TLX) ● 2 labels/participant: physical (every 5 min) and mental (pre/post-task) ● Monitoring: 25 min physical (manual handling) + 25 min mental (digits task), repeated over two days	● Controlled environment limits generalizability to actual construction sites ● Small sample size affects robustness and scalability
(Velmovitsky et al., 2022 ) [41] Using apple watch ECG data for heart rate variability monitoring and stress prediction	33	❖ Apple Watch Series 6 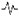 RMSSD, HRV, mean HR, SDNN (standard deviation of R-R intervals), and frequency-domain metrics (e.g., LF/HF ratio) derived from ECG	❖ Binary classification ● Classical ML, RF, and SVM ● 52% to 64% F1-weighted scores	IW	● Labels: self-reported stress (DASS-21; single-item Likert) ● 6 labels/participant daily (every 3 h; EMA) ● Monitoring: 2 weeks with Apple Watch (30 s ECG readings) ● Prompting: 6 times/day for ECG and stress questionnaires via mobile health platform	● Predominantly white female participants limit generalizability ● Real-life data collection introduces noise, reducing accuracy ● Models performed well for “no stress” but poorly for “stress” states
(Vila et al., 2019) [42] A real-life application of stress detection of passengers while traveling	1	❖ Empatica E4 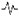 EDA/GSR, HR derived from BVP, ST, SC, and ACC	❖ Regression ● Personalized stress model (linear regression) ● 0.187 RMSE (raw output), 0.146 RMSE (Clipped Output), and 96.5% classification rate	IW	● Labels: self-reported stress (1–10 scale) ● 4 labels/participant (3 high-stress; 1 low-stress episode) ● High-stress: 162 min; low-stress: 120 min (museum visit) ● Monitoring: 3-day continuous monitoring while traveling; self-reports given daily	● Simple linear regression used without feature selection ● Small number of non-stress labels
(Weerasinghe et al., 2023) [43] Mental stress classification and exploring links between perceived and acute stress	22	❖ SynAmps amplifier and a 62-channel QuickCap EEG system 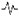 EEG signals from FP1, FP2, T7, and T8 channels	❖ Multiclass classification ● Spiking Neural Networks, Spike Time Dependent Plasticity (STDP), Intrinsic Plasticity (IP), Neuron Evolving and Pruning, self-pruning, unsupervised learning ● 90.76% accuracy	IL	● Labels: self-reported mental stress (PSS-14) ● 3 labels/participant (stress, neutral, and positive states) ● Monitoring: EEG monitoring across 3 sessions (2 min each) with 40 audio comments (10–15 sec each)	● Reliance on EEG data limits generalizability to other stress scenarios ● Potential overfitting to specific audio stress cues ● Spiking neural network model complexity hinders real-time wearable applications without optimization
(Xu et al., 2024) [44] Non-invasive stress monitoring using an electronic skin	10	❖ CARES sensor (electronic skin) 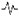 Pulse waveform, GSR/EDA, ST, and molecular biomarkers in sweat (glucose, lactate, uric acid, sodium, potassium, and ammonium)	❖ Multiclass classification ● Supervised learning, Shapley additive explanation ● 99.2% accuracy for stress/relaxation detection	IL and IW	● Labels: self-reported state anxiety (STAI-Y) ● 3 labels/participant (pre-, during, and post-stressor tasks) ● Monitoring: 24 h continuous monitoring across daily activities (exercise, lab work, and relaxation)	● CARES device promising but has research gaps ● Sweat cross-reactivity may reduce sensor accuracy ● Scalability and cost pose challenges ● Long-term wearability needs testing for skin compatibility ● ML algorithms may introduce bias and lack generalizability ● Real-world validation and data privacy are crucial
(Zhang et al., 2023) [45] Model the relationship between overcrowding and stress levels	26	❖ Empatica E4, Zephyr Bioharness 3, and FrontRow wearable camera 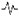 GSR/EDA, ST, and ECG	❖ GLMM analysis ● Mask R-CNN for image detection/Geographically weighted regression and GLMM for effects estimate; personalized models	IW	● Labels: self-reported stress via eDiary app ● 3 labels/participant (pre-, during, and post-walk) ● Locations: green, blue, transit, and commercial spaces ● Monitoring: 80 min (20 min per location: 5 min sitting; 15 min walking)	● Small sample size from a single setting limits generalizability ● Confounders like cultural and socio-economic differences not accounted for

**Figure 4:**
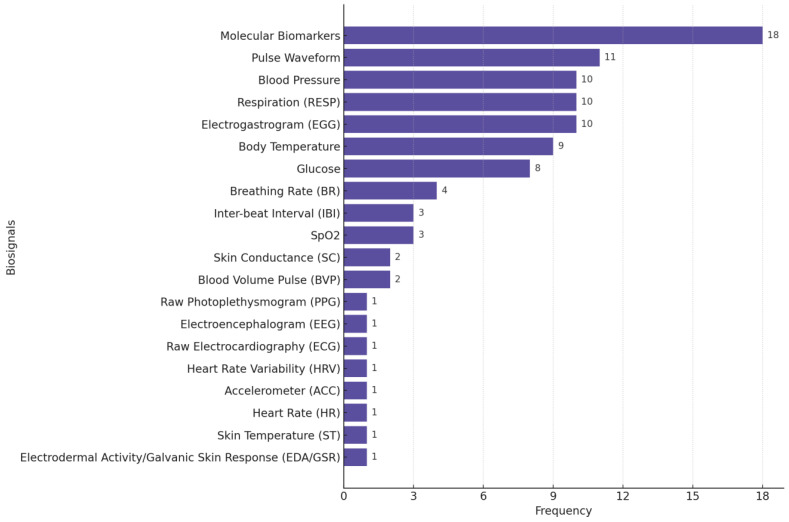
Distribution of biosignals used in stress-focused studies. Many studies used multiple biosignals.

Figure 4 illustrates the distribution of biosignals used in the stress monitoring studies we reviewed, highlighting the predominance of GSR/EDA, raw PPG, ST, HR, ACC, and HRV as common physiological signals used in stress monitoring/detection research.

A few studies highlighted battery drainage issues as a major barrier to real-world data collection. For example, Can et al. (2019) [24] used Samsung smartwatches for physiological data collection. However, the limited battery life of up to 4 h with all sensors posed a major challenge, requiring frequent recharging during the study. Similarly, Zhang et al. [45] reported rapid battery drainage of wearable cameras in cold temperatures, restricting data collection to daytime hours and highlighting environmental factors as a limitation for device performance.

Among the 29 studies reviewed in the field of stress, 21 were conducted with a small sample size. A total of 9 studies (out of 29) conducted their experiments in real-world conditions, while the remaining studies were performed under controlled laboratory settings.

**Figure 5:**
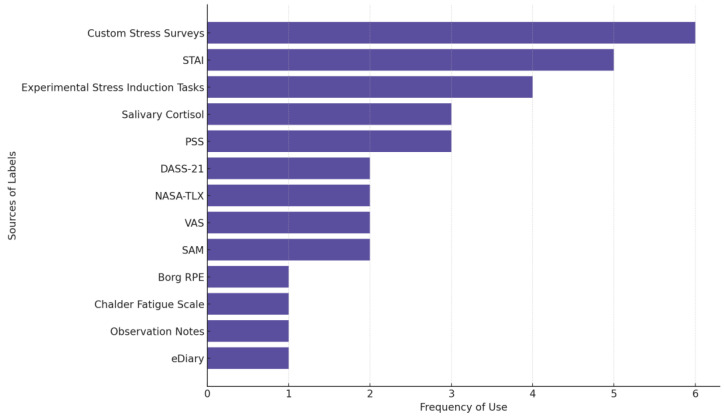
The source of labels in stress-focused studies. Some studies used multiple measures.

Figure 5 shows the most frequently used sources of stress labels across studies. Custom stress surveys are used in 6 of the 29 reviewed stress studies. These surveys are non-standardized, study-specific self-reports used to capture perceived stress levels, often through Likert scales (e.g., 1–5; 0–10), single-item stress measures, or tailored questionnaires. STAI (five studies) is the most used established questionnaire followed by PSS and measuring salivary cortisol levels as an outcome (three studies each).

We observed a variety of experimental stress induction methods, including cognitive stressors (e.g., Stroop Test; Mental Arithmetic), social stressors (e.g., Trier Social Stress Test; Maastricht Acute Stress Test), and other challenges (e.g., exams, puzzles, and physical exertion). These studies typically classify stress as a binary variable (e.g., stressed vs. not-stressed) and validate responses using physiological data [46,47].

### 3.2. Depression

**Figure 6:**
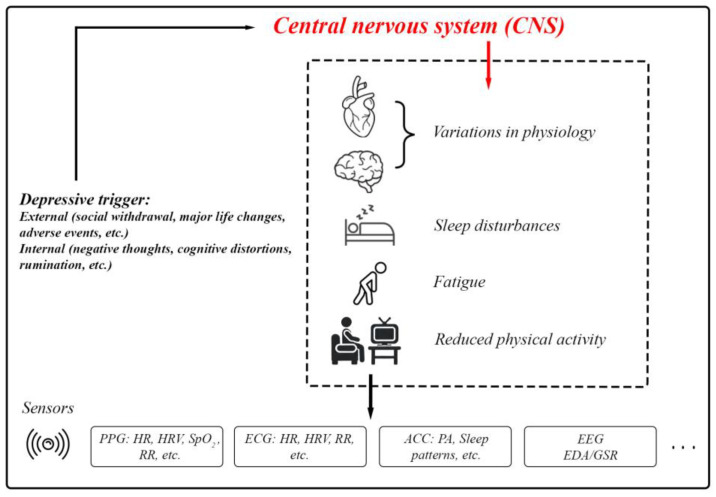
The body’s physiological responses to depression and the standard biosignals used for measuring these responses.

Depression typically arises from a complex interplay of social, biological, and psychological factors [48]. Depression is a core symptom of at least two pervasive mental health disorders: Major Depressive Disorder (a form of clinical depression that includes major depressive episodes) and Bipolar Disorder (includes a presentation of hypomanic/manic symptoms and often depressive episodes) [49,50]. Depressive episodes must last for at least two weeks for a clinical diagnosis and are characterized by persistent feelings of guilt, worthlessness, helplessness, changes in appetite and sleep patterns, suicidal thoughts, and a loss of interest in previously enjoyed activities [51]. Depression involves disruptions in the Central Nervous System (CNS). External and internal depressive triggers activate pathways that result in physiological, cognitive, and behavioral changes (Figure 6). These include variations in HR, neural activity, sleep disturbances, and fatigue. Sensors such as PPG, ECG, accelerometers, EEG, and EDA/GSR provide a means of quantifying these changes for research and clinical purposes [52,53,54,55,56].

In Table 3, we summarize the key findings from each study we reviewed involving the monitoring of depression using biosensors.

**Table 3:** Biosensing papers reviewed focusing on depression.

Article/Year Application	Sample Size	Wearable/ Feature Space	Prediction Task/ AI Details	Env.	Labels/ Monitoring Details	Research Gaps/ Future Challenges
(Chikersal et al., 2021) [57] Detecting and predicting depression in college students, with predicting symptoms up to 15 weeks in advance	138	❖ Fitbit Flex 2 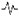 Steps, sleep metrics, Bluetooth (social proximity), calls, location, and phone usage	❖ Binary classification ● Personalized model ● 85.7% accuracy in detecting post-semester depressive symptoms, predicting these outcomes with accuracy >80%	IW	● Labels: pre- and post-semester depression severity scores using the Beck Depression Inventory-II (BDI-II) ● 2 labels/participant ● Monitoring: 16-week semester	● Passive data may miss nuances of depressive symptoms ● College student focus limits generalizability ● High dimensionality and small sample size challenge feature stability ● Refinement needed for missing data and robustness
(Dai et al., 2022) [58] Personalized depression predictions in RCTs	89	❖ Fitbit Alta HR 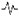 HR, sleep metrics, and physical activity (e.g., sedentary minutes, lightly active minutes, and fat burn minutes)	❖ Multitask Learning ● Multitask Learning, Hierarchical Model Architecture, Dynamic Task Weighing ● 0.725 AUROC; 0.668 AUPRC	IW	● Labels: depression remission outcomes based on PHQ-9 scores ● 2 labels/participant (baseline and after 6 months) ● Monitoring: 6 months ● Continuous monitoring via Fitbit and assessments at 2, 4, and 6-month check-ins	● Assumes static treatment paths post-randomization, limiting use in adaptive trials ● Small sample size reduces confidence in results ● The impact of varying wearable data lengths on model performance was not evaluated
(Kim et al., 2019) [59] Classify depression in older adults living alone	47	❖ Actiwatch Spectrum PRO (Philips Respironics) 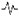 Sleep efficiency, physical activity, and ambient light exposure	❖ Binary classification ● Binary LR compared with 4 ML models (logit, DT, boosted trees, and RF) ● ~70% binary LR accuracy; 91% logit model accuracy	IW	● Labels: self-reported depression (EMA via Actiwatch, SGDS-K, and K-HDRS) ● 4 EMA prompts/day + 2 SGDS-K and K-HDRS labels (baseline; 2 weeks) ● Monitoring: 2 weeks (56 labels/participant) ● Prompting: 4 times/day (10-point Likert scale)	● Subjective reports limit accuracy without clinical diagnoses ● EMA grand means may miss moment-to-moment mood variations ● Small, predominantly female sample limits generalizability ● Low statistical power hinders detecting sleep efficiency associations
(Li et al., 2020) [60] Recognizing mild depression	51	❖ HydroCel Geodesic Sensor Net (EEG system with 128 channels) 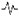 EEG signals from delta, theta, alpha, beta, and gamma frequency bands	❖ Binary classification ● Used different models including CNN, SVM, and KNN ● 80.74% accuracy (highest)	IL	● Labels: BDI-II scores classify participants as mildly depressed or healthy ● 2 labels/participant (mildly depressed; healthy) ● Monitoring: EEG monitoring during a 7 min facial expression task	● Focus on mild depression limits applicability to more severe cases ● Heavy reliance on EEG data quality and preprocessing may affect robustness and generalizability ● Small, homogeneous sample from a single university limits broader applicability
(Mullick et al., 2022) [61] Predicting adolescent depression during COVID-19, with personalized models	37	❖ Fitbit Inspire HR 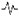 HR, sleep (duration and quality), steps, calls, conversations, locations, screen usage, and Wi-Fi, from mobile devices and Fitbit	❖ Multiclass classification ● Algorithms: LASSO, elastic net, RF, AdaBoost, extra trees, gradient boosting, and XGBoost; high PHQ-9: 60–75% accuracy; low PHQ-9: 20–40% accuracy ● 50–60% average accuracy	IW	● Labels: self-reported depression levels using the PHQ-9, completed weekly by participants ● 24 labels/participant (one per week) ● Monitoring: 24 weeks	● COVID-19 impact limits generalizability to non-pandemic conditions ● Missing data from technical issues and non-adherence affected accuracy ● Small sample size reduced robustness despite study duration ● Model struggled with rare/severe depression changes, needing better data handling
(Pedrelli et al., 2020) [62] Monitoring changes in depression severity	31	❖ Empatica E4 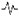 EDA/GSR, HRV, ST motion (ACC), sleep metrics, phone usage, call and messaging behavior, app usage, location change patterns, and activity levels	❖ Regression task (depression severity) ● Average ensemble of boosting and RF ● Correlation between models’ estimate and clinician-rated HDRS scores: 0.46–0.7	IW	● Labels: HDRS-17 assessed by clinicians during in-person visits ● 6 labels/participant (assessed bi-weekly for 8 weeks) ● Monitoring: 9 weeks	● Data collection issues require better network and sensor connectivity ● High MAE (3.8–4.74) limits clinical scalability despite strong correlations ● Small sample size and low symptom variability reduce generalizability ● Minimal sleep impact due to short study; longer monitoring needed
(Price et al. 2022) [63] Unexpected similarities found between schizophrenia patients and healthy controls	77	❖ Actiwatch device 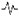 Minute-by-minute physical activity levels	❖ UMAP/SHAP ● Unsupervised ML clustering methods. An unsupervised ML algorithm	IW	● Labels: actigraphy-based movement patterns and clinical diagnoses ● 2 labels/participant (schizophrenia, depression, or control) ● Monitoring: 1-week continuous monitoring with actigraphy devices	● Single-institution sample limits generalizability ● Cross-group differences may be confounded by age, medication, disorder type, and gender ● Depression and schizophrenia severity levels may not reflect typical cases
(Rykov et al., 2021) [64] Predicting workforce depression	267	❖ Fitbit Charge 2 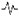 Steps, HR, sleep metrics, circadian rhythm metrics (e.g., inter-daily stability and autocorrelation)	❖ Binary classification ● Dropouts meet Multiple Additive Regression Trees ● 80% accuracy, 82% sensitivity, and 78% specificity	IW	● Labels: depressive symptom severity assessed using the 9-item PHQ-9 ● 2 labels/participant (PHQ-9 scores at baseline and after 14 days) ● Monitoring: 14 days	● Workforce-specific cohort limits generalizability ● Findings apply mainly to balanced demographic subgroups; broader testing needed ● Self-reported depression assessments may introduce response bias
(Sato et al., 2023) [65] Enhanced MDD screening using SQIs to filter motion artifacts	69	❖ Empatica E4 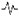 HRV derived from PPG and ACC for activity and sleep detection	❖ Binary classification ● Classical ML; linear classification model ● 87.3% sensitivity; 84.0% specificity	IW	● Labels: depressive symptoms using the Zung Self-Rated Depression Scale (SDS) ● 2 labels/participant (self-reported SDS scores and physiological markers over 24 h) ● Monitoring: 24 h	● Small sample (69 participants) limits generalizability ● Linear models may miss complex HRV patterns ● Wearable device variability not fully assessed, affecting HRV accuracy
(Bai et al., 2022) [66] Tracking mood stability and predicting variations in MDD for personalized treatment	261	❖ Mi Band 2 (Xiaomi Corporation) and phone usage statistics 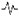 Call logs, sleep data, step count data, and HR	❖ Binary/multiclass classification ● SVMs, KNN, DT, NB, RF, and LR ● RF with 84.46% accuracy and 97.38% recall for predicting between Steady-remission and Mood Swing-moderate	IW	● Labels: PHQ-9 assessments ● Each participant contributed three consecutive PHQ-9 results per data sample ● Monitoring: 12 weeks ● Prompting: Daily at 8 PM to record mood using the Visual Analog Scale (VAS)	● Imbalanced, small dataset affects model generalizability and accuracy ● Restricted to Android users, excluding a significant population segment
(Shah et al., 2021) [67] Depression management	14	❖ Samsung Galaxy watch 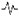 HR, steps, and exercise metrics (e.g., speed, calories, distance), exercise data, sleep duration, and quality, HRV metrics derived from PPG	❖ Regression task ● SHAP used to compare ML models per subject (RF, gradient boost, AdaBoost, elastic net, SVM, and Poisson regressor) ● Voting regressor selected the best model from all strategies ● Spearman’s rho = 0.67, *p* < 0.0001	IW	● Labels: self-reported mood (EMA, 7-point Likert for depression/anxiety) ● 4 labels/participant daily (8 a.m., 12 p.m., 4 p.m., and 8 p.m.) for 30 days ● Monitoring: 1 month ● Prompting: 4 times/day via the EMA app to report mood, lifestyle factors, and stress levels	● N-of-1 models’ treatment guidance remains unproven ● Low-variability participants led to poor model fits ● Wearables and EEG may lack comprehensive data capture ● Small, specific sample limits generalizability ● Lack of clinical interviews weakens depression assessment ● High-frequency data collection may impact long-term adherence and quality
(Tazawa et al., 2020) [68] Assessing depression severity and mood disorder evaluation	86	❖ Silmee W20 (TDK, Inc., Tokyo, Japan) wristband biosensor device 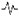 HR, ST, step count, energy expenditure, body movement, sleep time, and UV light exposure	❖ Binary classification/regression ● XGBoost, self-rating assessment score (labels) ● 76% accuracy, 73% sensitivity, and 79% specificity	IW	● Labels: clinician-administered HAMD-17 and self-reported BDI-II ● 2 labels/participant (HAMD-17 depressive severity; BDI-II depression score) ● Monitoring: continuous monitoring for up to 7 days	● Small, non-diverse sample limits generalizability and significance ● No external validation; medication and hospitalization effects excluded
(Tian et al., 2023) [69] Diagnosing depression	178	❖ The three-lead EEG sensor and electrodes 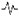 EEG signals from three prefrontal lobe electrodes (Fp1, Fpz, and Fp2)	❖ Binary classification ● Using different classification models including “KNN, SVM, NB, DT, RF, XGboost” ● 90.70% accuracy, 96.53% specificity, and 81.79% sensitivity (KNN)	IL	● Labels: clinician-administered assessments using the PHQ-9 for depression ● 2 labels/participant ● Monitoring: 162 s, including 90 s of resting-state EEG data and 72 s of audio-stimulated EEG data	● Small, specific sample limits generalizability ● ALO algorithm’s complexity may hinder real-time clinical use ● Further validation needed across diverse populations ● Three EEG leads may limit detection versus multi-lead systems
(Yang et al., 2023) [70] Depression recognition using multimodal data for improved severity assessment	57	❖ Custom wristband with audio, activity (gyroscope; accelerometer), and heart rate sensors 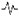 Features: audio (energy, entropy, formants, and brightness), activity (acceleration; angular velocity), and HR from PPG	❖ Binary classification ● Using different models XGBoost RF, DT, LR, KNN, and NB (Gaussian) ● 83% accuracy	IW	● Labels: self-reported depressive symptoms using multiple questionnaires, including the PHQ-9, BDI, and the Center for Epidemiological Studies Depression (CES-D) scale ● 3 labels/participant, collected before and after a course of treatment ● Monitoring: one day at a time (from 8:00 A.M. to 10:00 P.M.)	● Larger sample needed for validation across populations ● Long-term effectiveness in real-world settings untested ● Emotion-sensing graphs and GCN model may hinder real-time use due to high computational demands
(Zhu et al., 2019) [71] Mild depression detection during free viewing	51	❖ 128-channel HydroCel Geodesic Sensor Net (EEG) and EyeLink 1000 Desktop Eye Tracker 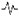 EEG and EM data were synchronized using the TTL signal method for precision	❖ Binary classification ● Models: Linear SVM, Radial Basis Function SVM (RBF SVM), Gradient Boosting Decision Tree (GBD tree), RF, Self-Normalizing Neural Networks (SNNs), and Batch Normalized Multilayer Perceptron (BNMLP) ● 83% accuracy (highest)	IL	● Labels: self-reported depressive symptoms (BDI-II) ● 2 labels/participant (mild depression or normal control) ● Monitoring: 30 trials, with each trial lasting 6 s of viewing neutral and emotional facial expressions, followed by 2 s of rest, for a total of 240 s of active monitoring per participant	● Limited sample size affects generalizability with only two depression levels studied ● Excluded severe depression, requiring broader diagnostic capabilities ● Inconsistent EEG band performance with EM, needing optimization in feature selection and fusion strategies

**Figure 7:**
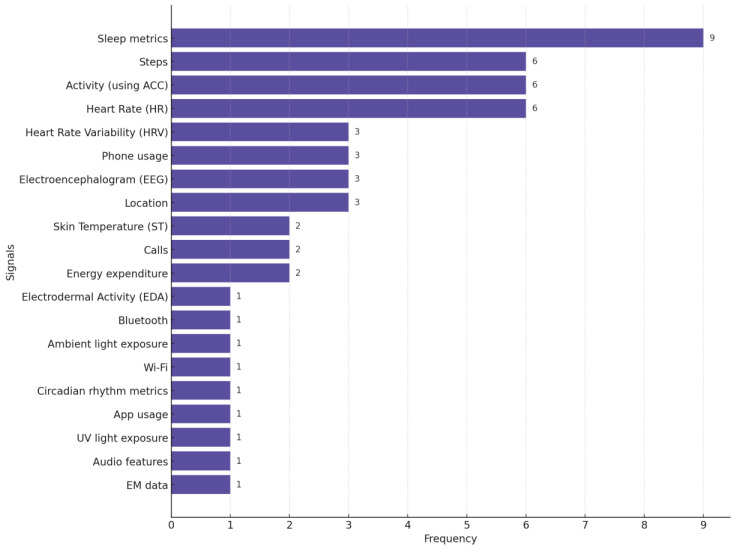
Distribution of biosignals used in depression-focused studies. Many studies used more than one biosignal.

In Figure 7, we provide the distribution of different biosignals used in studies focused on depression monitoring. This chart highlights the pervasiveness of sleep metrics, steps (the number of steps participants walk or run daily), activity, and HR as primary biosignals for detecting and monitoring depression.

In the reviewed depression research, most of the studies were conducted using medium-to-large sample sizes, with only 1 study (out of 15) using a small sample size. Three studies were conducted in controlled laboratory settings.

Several studies reported battery drainage issues as a limitation when using wearable devices for data collection. Bai et al. [66] noted that Android system restrictions prevented their app from running continuously in the background, affecting data collection continuity. Mullick et al. [61] observed lower adherence rates with Fitbit devices due to frequent charging requirements and occasional participant forgetfulness. Similarly, Pedrelli et al. [62] highlighted that adherence issues arose with the E4 Empatica wristbands, which required participants to wear the devices for 22 h daily, reserving 2 h for charging and data uploads. Yang et al. [70] reported battery constraints in their custom-developed wristbands, which were equipped with lithium batteries that allowed continuous use only between 8:00 AM and 10:00 PM daily.

**Figure 8:**
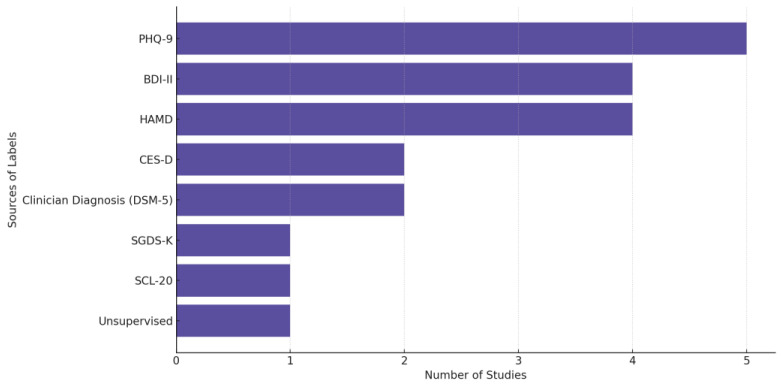
Sources of the labels used in depression-focused studies.

In Figure 8, we provide the distribution of label sources used across depression-related biosensing studies, highlighting the predominant reliance on structured clinical scales such as PHQ-9, HAMD, and BDI-II, alongside clinician diagnoses guided by DSM-5. “Unsupervised” refers to studies that did not rely on predefined diagnostic labels but instead applied unsupervised learning techniques to identify patterns or clusters in the data without explicit supervision from clinician assessments or self-reports. We observe a preference in the field for using validated assessment tools and standardized diagnostic criteria in labeling depression-related data.

### 3.3. Anxiety

Anxiety disorders (e.g., Generalized Anxiety Disorder and Social Anxiety Disorder) are characterized by persistent and excessive fear and worry and are often accompanied by physical symptoms such as chest pain, headaches, rapid heartbeat, and abdominal discomfort [72]. An epidemiological survey conducted in 2018 reported a global prevalence of anxiety disorders ranging from 6% to 16% [73]. Social isolation, difficulty working, restlessness, and challenges in communication are major problems faced by individuals with anxiety [74].

Table 4 provides a summary of the key findings from each study we reviewed on AI-powered biosensing for anxiety.

**Table 4:** Biosensing papers reviewed focusing on anxiety.

Article/Year Application	Sample Size	Wearable/ Feature Space	Prediction Task/ AI Details	Env.	Labels/ Monitoring Details	Research Gaps/ Future Challenges
(Jacobson et al., 2021) [75] Predicting symptom deterioration in GAD and PD for early intervention	265	❖ Mini Mitter Actiwatch 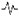 Activity counts (total, average, and max), sleep patterns (latency, WASO, efficiency, and total time), and circadian rhythm irregularities	❖ Binary classification ● Unsupervised deep autoencoder with ensemble modeling ● Base learners: neural networks, splines, ridge regression, RF, GLMs, Gaussian processes, XGBoost, KNN, and SVM ● 0.696 AUC, CI [0.598, 0.793], 84.6% sensitivity, 52.7% specificity, and 68.7% balanced accuracy	IW	● Labels: anxiety disorder symptoms assessed via the Composite International Diagnostic Interview (CIDI) ● 2 labels/participant (baseline and after 17–18 years) ● Monitoring: one week for actigraphy data collection, with follow-up interviews 17–18 years later	● Specificity of 52.7% risks false positives and unnecessary interventions ● Sparse data collection over two decades may miss symptom fluctuations, affecting accuracy ● Low prevalence of GAD and PD limits generalizability
(Lee et al., 2022) [76] Identifying geriatric depression and anxiety	352	❖ Fitbit Alta HR2 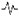 HR, steps, sleep-related metrics (sleep onset latency, wake after sleep onset, sleep quality, and total sleep time) and circadian rhythm metrics (e.g., inter-daily stability, intra-daily variability, and dominant rest phase onset)	❖ Multilabel classification ● Classical ML LR, SVM, RF, and gradient boosting (GB) ● 90% accuracy for single labeling	IW	● Labels: self-reported depression and anxiety symptoms using the Korean version of the Geriatric Depression Scale (K-SGDS) and the Korean Geriatric Anxiety Inventory (K-GAI) ● 2 labels/participant (depression; anxiety) from questionnaires ● Monitoring: 1+ month using low-cost activity trackers	● Moderate-resolution trackers may lower data quality vs. ActiGraph ● Binary relevance method overlooks depression–anxiety relationships ● Seasonal mood and activity variations not considered ● More data needed to reduce prediction variance
(Shaukat-Jali et al., 2021) [77] Detecting social anxiety and its levels	12	❖ Empatica E4 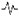 EDA/GSR, HR, and ST	❖ Binary (non-anxious vs. socially anxious) and multiclass (baseline, anticipation, and reactive anxiety) classification ● Supervised learning ● Accuracy: 97.5–99.48% (binary); 98.86–99.52% (multiclass with severity) with EDA/GSR being the most predictive physiological marker	IL	● Labels: self-reported subclinical social anxiety levels using the Liebowitz Social Anxiety Scale (LSAS-SR) and the Social Phobia Screening Questionnaire (SPSQ) ● 3 labels/participant (baseline, anticipation anxiety, and reactive anxiety) ● Monitoring: approximately 30 min during the task	● Small sample size limits generalizability ● Models may be biased toward certain classes, inflating accuracy ● Uncontrolled external factors, such as caffeine and alcohol consumption, may affect results
(Di Tecco et al., 2024) [78] Detecting anxiety induced by a horror movie trailer	34	❖ Shimmer3 GSR+ 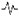 GSR/EDA, PPG, and HR	❖ Multiclass classification ● Classifiers based on DT, Discriminant Analysis, NB, SVM, ANNs, KNN, Kernels, and Ensembles optimized using Bayesian optimizer ● > 95% accuracy	IL	● Labels: self-reported anxiety (post-task questionnaires) ● 3 labels/participant (baseline, anxiety-inducing, and relaxation) ● Monitoring: 10 min monitoring (2 min horror trailer; 4 min relaxation clips before/after)	● Limited audiovisual stimuli reduce specificity and applicability ● Improved data collection and signal processing needed for precision and reliability

HR, used in three of the reviewed studies, is the most commonly used biosignal for measuring anxiety out of the included papers, followed by EDA/GSR and sleep metrics, each appearing in two studies. ST, PPG, circadian rhythm, and activity levels are each used in one study. Due to the small number of studies in this category, we do not include a biosensor distribution figure for anxiety studies.

None of the studies that focused on the anxiety domain explicitly reported significant battery drainage issues.

The biosensing studies focused on anxiety used a diverse array of clinical tools to source labels for anxiety and related conditions. Jacobson et al. [75] trained models using two distinct labeling approaches to predict long-term anxiety symptom deterioration: (1) passive actigraphy data collected over a week, capturing movement and sleep parameters (e.g., wake time, sleep efficiency, and number of wake bouts), and (2) self-reported anxiety symptoms measured via the Composite International Diagnostic Interview (CIDI) at baseline and follow-up, along with five Likert-scale anxiety indicators (e.g., nervousness; jitteriness) collected during a daily diary study. Lee et al. [76] relied on the Korean Short Form of the Geriatric Depression Scale (K-SGDS) and Korean Geriatric Anxiety Inventory (K-GAI) to measure depression and anxiety. Shaukat-Jali et al. [77] used the Liebowitz Social Anxiety Scale (LSAS-SR) and predefined experimental phases to label social anxiety states. Di Tecco [78] et al. derived labels from participant-reported emotions during an anxiety-inducing video experiment. These tools combine structured diagnostics with subjective self-reports for labeling. Because each study used a separate scale, we do not include a figure for anxiety scales.

### 3.4. General Trends

**Figure 9:**
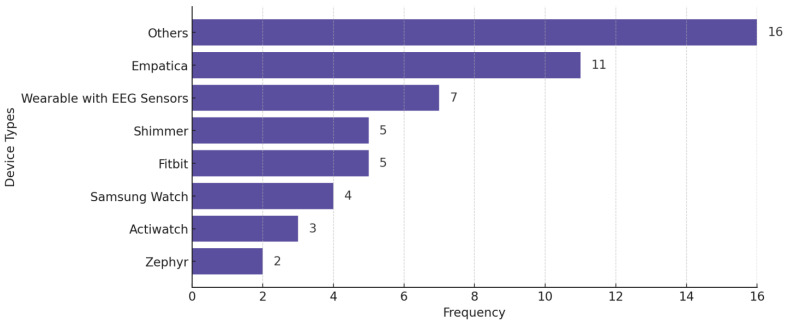
Wearable devices used in the reviewed studies across all mental health conditions. Some studies used more than one wearable.

**Figure 10:**
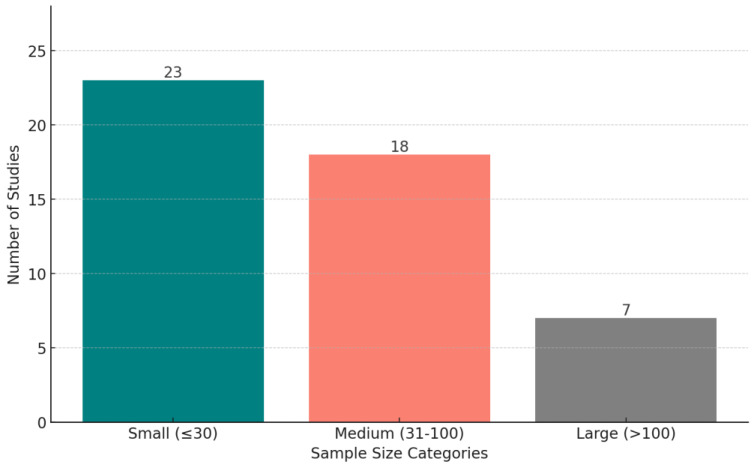
The distribution of studies based on sample size categories: small (fewer than 30 participants), medium (30–100 participants), and large (more than 100 participants).

Figure 9 shows the distribution of wearable devices used in the reviewed studies, across all conditions. The criterion for explicit categorization is that a device must appear in at least two studies—otherwise, we put the device in the “Others” category. Figure 10 displays the distribution of studies categorized by sample size, highlighting the number of studies with small (<30 participants), medium (30–100 participants), and large (>100 participants) datasets.

## 4. Risks of Bias and Applicability Appraisal

**Figure 11:**
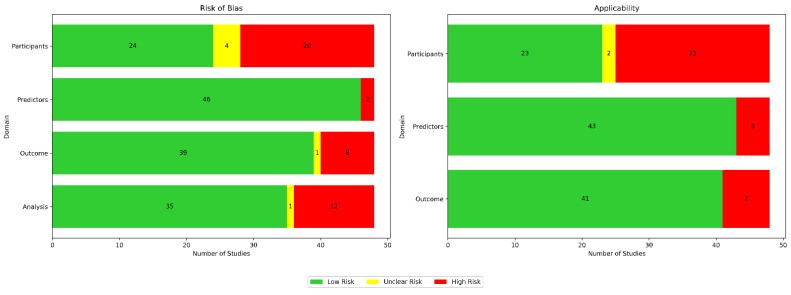
Appraisal of bias using PROBAST. Green indicates low risk, red indicates high risk, and yellow denotes unclear risk.

Using the Prediction Model Risk of Bias Assessment Tool (PROBAST) guidelines, we assessed the risk of bias and applicability for all 48 papers across four domains for bias (participants, predictors, outcomes, and analysis) as well as three domains for applicability (participants, predictors, and outcomes), as illustrated in Figure 11. In Table 5, we provide a breakdown of the risk of bias (ROB) assessments per paper.

Bias in terms of participants was usually attributed to small sample sizes and homogeneous data sources. Bias in terms of predictors was usually due to either inconsistent measurements when different wearable devices were used, sensor noise, or missing data. Bias in terms of outcomes was usually due to bias from self-reported labels and because assessments were conducted under specific, controlled conditions. Bias in terms of analysis was usually due to the use of small and unrepresentative samples. Applicability concerns in terms of participants were usually due to insufficient information about participant recruitment or inclusion/exclusion criteria. Applicability concerns regarding predictors were usually due to the use of medical-grade wearables, the inclusion of multiple types of wearable devices in a single study, or the inherent limitations of the used wearable technology. Applicability concerns regarding outcomes typically arose due to strict protocols in controlled environments not reflecting real-life scenarios.

## 5. Discussion

### 5.1. Key Challenges for the Field

We have conducted a systematic review on the integration of biosensing and AI for mental health sensing. We have gleaned several trends in the field that highlight ongoing challenges and corresponding opportunities:

**Lack of ecological validity:** although our inclusion criteria focused on studies utilizing wearable devices capable of passive data collection, we found that many of these studies did not fully leverage the potential for continuous, real-world monitoring. A significant number of studies in the stress domain (20 out of 29), for example, collected data in controlled laboratory settings during short-term experiments. This suggests that while wearables offer the capability for continuous passive data collection, their application has often been limited to short-duration, controlled environments rather than extended real-world contexts, thus limiting the ecological validity of the findings of these studies.

There are many challenges that arise in real-world settings. For example, participants may engage in various activities simultaneously, such as moving between different locations or interacting with their environment, which can introduce noise and confounders into the data. These uncontrolled factors, including changes in posture, physical activity, or device placement, can impact the reliability of the collected signals for specific analyses, complicating the monitoring process and potentially reducing the effectiveness of predictive systems for specific mental health conditions. In order to improve data quality and validity from in-the-wild samples, we recommend innovations that can align the timing of ecological momentary assessment (EMA) prompts (i.e., patient-reported outcomes such as mental or emotional states provided in real-time or near-real-time, often prompted at randomized or scheduled intervals throughout the day [79]) with moments of uncertainty in the collected data, for example, by using active learning techniques that optimize timing based on the confidence of machine learning models. By using uncertainty-based active learning approaches, the system can request input from participants only when the ML model is least confident in its predictions. Such approaches can improve just-in-time adaptive interventions (JITAIs) [80,81], which leverage machine learning models to optimize the timing of EMA prompts. Additionally, automated protocol compliance systems can verify whether wearables are being used correctly and whether longitudinal passive data collection procedures are continuously successful. Such systems can monitor device performance and use persistent reminders to ensure that participants remain compliant with the data collection protocol, reducing instances of missing or low-quality data [82,83].

**Small sample sizes:** many studies were constrained by small sample sizes (23 out of 48) and a lack of demographic diversity, limiting the generalizability of findings to the broader population. For example, the predominance of young, well-educated participants in some studies may not reflect the experiences of older adults, children, or individuals from under-represented socio-economic backgrounds. Research has shown that stress, depression, and anxiety can vary significantly across these groups; for instance, older adults may experience stress and depression due to health-related challenges, while younger populations may face stress linked to academic or career pressures [84,85]. Similarly, individuals from disadvantaged socio-economic backgrounds may be more vulnerable to chronic stress due to financial instability or lack of access to mental health care [85,86]. Future research should prioritize expanding sample sizes and including more diverse populations to better capture these nuanced differences and ensure the broader applicability of predictive models for mental health.

**Low performance due to data heterogeneity:** the heterogeneity of biosensing data remains a central challenge for developing robust AI models and is likely a key contributor to the low performances we observed across several of the reviewed papers. One promising, previously proposed area of future research that can address this issue is personalized machine learning, where a separate biosensing AI model is trained for each individual [87,88]. A few emerging works have explored the use of personalized self-supervised learning to enable biosensing deep learning models to learn personalized feature extractors from unlabeled signals, often [89,90,91], though not always [92], improving performance over baseline approaches that do not incorporate personalized self-supervised pre-training. However, personalized self-supervised learning can fail to achieve performance gains over purely supervised personalized learning in challenging real-world settings where perfect participant compliance is rare, such as when predicting subjective outcome measures from data collected during work hours [92]. This highlights a central opportunity for the field to innovate in human–computer interaction innovations that enable more seamless data collection procedures in challenging environments. Furthermore, there is a need for advanced data processing techniques to handle missing, noisy, and imbalanced data, which are common challenges in the real-world settings that AI-driven biosensing tends to occur in. Finally, it is important to ensure that data from diverse physiological sensors are well integrated with personalized baselines through model calibration. The development of robust algorithms that account for these factors while maintaining high performance will be crucial for translating AI biosensing systems to the real world.

**Power efficiency and miniaturization in wearable biosensors:** several of the studies we reviewed noted issues with power consumption. Miniaturization of wearable biosensors can enhance user comfort but poses challenges for power management. Smaller devices have limited space for batteries, which can restrict operational time and sensing capabilities. To address this, researchers are developing energy-efficient strategies that aim to balance device compactness with sufficient power for continuous health monitoring. For example, Taji et al. [93] integrated low-power microcontrollers and optimized data acquisition through an energy-efficient frequency selection technique, reducing power consumption and extending battery life. Wang et al. proposed adaptive duty cycling, which dynamically adjusts sensor configurations based on user activity, reducing power consumption by approximately 69% while maintaining activity recognition accuracy [94,95].

### 5.2. Limitations

There are important limitations of this review. First, we did not study every possible mental health condition, limiting our scope to only stress, anxiety, and depression. Second, our inclusion and exclusion criteria eliminated studies that did not specifically use wearable devices capable of passive data collection or relied on publicly available datasets. Third, our systematic search strategy was intentionally focused on stress, anxiety, and depression due to their high prevalence and significant impact on global mental health. It would have been infeasible to review every possible health condition. However, it is possible that a review with an expanded or different scope that includes additional or alternate conditions, such as bipolar disorder or schizophrenia, would have yielded additional or differing insights. Fourth, this review includes studies that use a variety of wearables and sensors, which introduces variability in the reviewed data collection methods, sensor qualities, and processing techniques. This heterogeneity may have contributed to inconsistencies across findings and reduced our ability to directly compare results across studies.

## 6. Conclusions

We systematically reviewed AI-powered biosensing applications for mental health. Based on our findings, we recommend that future studies in this field aiming for high impact should (1) focus on gathering a large sample size outside of the research lab, (2) improve ecological data quality and validity through automated algorithms to ensure protocol compliance, and (3) personalize models to address between-subject heterogeneity of biosignals.

## Figures and Tables

**Table 1:** Query performed on the Web of Science database and the resulting number of unique papers reviewed, categorized by the publisher.

Search Term	Publisher	No. of Papers
((“sensing” OR monitor* OR track* OR measur* OR biosignal OR biomarker OR ecg OR ppg OR eeg OR gsr OR “galvanic skin response” OR “breathing rate” OR “respiratory rate” OR accelerometer OR posture OR “heart rate” OR steps OR “step count” OR hrv OR “heart rate variability” OR “skin temp*”) AND (wearable* OR smartwatch OR “biometric trackers” OR “health monitoring devices” OR “activity track*” OR “fitness track*” OR “fitness monitor*” OR “body worn sensor*” OR “smart band*”) AND (mental* OR wellbeing OR stress OR anxiety OR substance OR depression) AND (“machine learning” OR “deep learning” OR “artificial intelligence” OR AI OR ML))	IEEE	13
	MDPI	11
	Elsevier	6
	JMIR	6
	Frontiers	3
	Others	9

**Table 5:** Risk of bias (ROB) and applicability (A) assessments for reviewed studies using the PROBAST framework.

Study	ROB-Participants	ROB-Predictors	ROB-Outcome	ROB-Analysis	A-Participants	A-Predictors	A-Outcome
[17]	high	low	low	low	high	low	low
[18]	high	low	low	low	low	low	low
[19]	low	low	low	low	high	low	low
[20]	low	low	low	high	high	low	low
[21]	low	low	low	unclear	high	low	low
[22]	low	low	low	low	low	low	low
[23]	low	low	low	high	low	low	low
[24]	low	low	high	low	low	low	low
[25]	low	low	low	low	high	low	low
[26]	low	low	high	low	low	low	high
[27]	low	low	low	low	low	low	low
[28]	low	low	high	low	low	low	high
[29]	high	high	low	low	high	low	low
[30]	unclear	low	low	high	high	low	low
[31]	low	low	unclear	low	high	low	high
[32]	unclear	high	low	low	low	low	low
[33]	high	high	low	high	high	high	low
[34]	high	low	low	low	high	low	low
[35]	unclear	low	low	low	unclear	low	low
[36]	high	low	low	low	low	low	low
[37]	high	low	high	high	high	low	high
[38]	high	high	high	high	high	high	high
[39]	high	low	high	high	high	low	high
[40]	high	low	low	low	high	low	low
[41]	high	low	low	low	high	low	low
[42]	high	low	high	high	high	low	high
[43]	low	low	low	low	low	low	low
[44]	high	low	low	low	high	low	low
[45]	high	low	low	low	high	low	low
[57]	high	low	low	low	high	low	low
[58]	low	low	low	low	low	low	low
[59]	high	high	high	high	low	low	low
[60]	low	low	low	low	low	low	low
[61]	high	low	low	high	high	low	low
[62]	low	low	low	low	low	low	low
[63]	high	low	low	high	high	low	low
[64]	low	low	low	low	low	low	low
[65]	low	low	low	low	low	low	low
[66]	low	low	low	low	low	low	low
[67]	low	low	low	low	low	low	low
[68]	low	low	low	low	low	low	low
[69]	low	low	low	low	low	low	low
[70]	unclear	low	low	low	unclear	low	low
[71]	high	low	low	low	high	low	low
[75]	low	low	low	low	low	low	low
[76]	low	low	low	low	low	low	low
[77]	high	low	low	high	high	low	low
[78]	low	low	low	low	low	low	low
